# Sprouty3 and Sprouty4, Two Members of a Family Known to Inhibit FGF-Mediated Signaling, Exert Opposing Roles on Proliferation and Migration of Glioblastoma-Derived Cells

**DOI:** 10.3390/cells8080808

**Published:** 2019-08-01

**Authors:** Burcu Emine Celik-Selvi, Astrid Stütz, Christoph-Erik Mayer, Jihen Salhi, Gerald Siegwart, Hedwig Sutterlüty

**Affiliations:** Institute of Cancer Research, Department of Medicine I, Comprehensive Cancer Center, Medical University of Vienna, A-1090 Vienna, Austria

**Keywords:** Sprouty proteins, brain cancer, FGF-mediated signaling, tumor suppressor, tumor promoter

## Abstract

Dysregulation of receptor tyrosine kinase-induced pathways is a critical step driving the oncogenic potential of brain cancer. In this study, we investigated the role of two members of the Sprouty (Spry) family in brain cancer-derived cell lines. Using immunoblot analyses we found essential differences in the pattern of endogenous Spry3 and Spry4 expression. While Spry4 expression was mitogen-dependent and repressed in a number of cells from higher malignant brain cancers, Spry3 levels neither fluctuated in response to serum withdrawal nor were repressed in glioblastoma (GBM)-derived cell lines. In accordance to the well-known inhibitory role of Spry proteins in fibroblast growth factor (FGF)-mediated signaling, both Spry proteins were able to interfere with FGF-induced activation of the MAPK pathway although to a different extent. In response to serum solely, Spry4 exerts its role as a negative regulator of MAPK activation. Ectopic expression of Spry4 inhibited proliferation and migration of GBM-originated cells, positioning it as a tumor suppressor in brain cancer. In contrast, elevated Spry3 levels accelerated both proliferation and migration of these cell lines, while repression of Spry3 levels using shRNA caused a significant diminished growth and migration velocity rate of a GBM-derived cell line. This argues for a tumor-promoting function of Spry3 in GBMs. Based on these data we conclude that Spry3 and Spry4 fulfill different if not opposing roles within the cancerogenesis of brain malignancies.

## 1. Introduction

The term brain cancer summarizes multiple subtypes of tumors originating from different tissues of the central nervous system [1]. The most prevalent type of brain tumors are gliomas which arise from glial or precursor cells. They include, among others, lower graded astrocytoma (AC) and oligodendroglioma (ODG), as well as the WHO Grade IV classified glioblastoma multiforme (GBM) and its variant gliosarcoma (GS). GBM are the most common brain tumors and patients have a poor prognosis with a five-year survival rate of only 5.6% [2]. A group of neuronal tumors arising in the central but also in the autonomic nervous system are the rare neuroblastoma (NB) which are the second most common tumors in children [3]. Like in all human cancer cells, malignant transformation in gliomas is driven by typical chromosomal changes. The Cancer Genome Atlas project identified alterations in the network regulated by receptor-tyrosine kinases (RTK) as a frequent molecular cause of these cancers. Important molecules responsible for transducing the signals like the epidermal growth factor receptor (EGFR), the phosphatidylinositol 3-kinases (PI3K), NRAS and BRAF are frequently altered to a more efficient state, while inhibitors of their activities like neurofibromin (NF1) and the Phosphatase and TENsin homolog (PTEN) are often deleted or less effective [4].

Sprouty (Spry) proteins which represent modulators of RTK-driven signaling pathways were first identified as inhibitors of fibroblast growth factor (FGF)-induced signaling in *Drosophila* [5]. In humans, four homologues were described [6]. In contrast to the other Spry family members which are ubiquitously expressed in all tissues [6], the Spry3 encoding gene localizes to the pseudoautosomal region 2 and its expression is rarely documented. Only in brain and glia, Spry3 expression is doubtless detected [7]. Spry proteins fulfill important functions in many RTK-mediated signal transduction cascades. Primarily, they are known to interfere specifically with MAPK-ERK activation [8,9,10], but in other systems they were shown to influence the PI3K pathway as well [11]. Additionally, Spry proteins are able to interfere with phospholipase C-induced pathways [12]. In contrast to their manifold inhibitory function on RTK-mediated pathways, Spry proteins are able to interact with the E3-ubiquitin ligase c-Cbl and thereby constrict the degradation of some RTKs as shown for the EGFR [13]. Considering their functions in fine tuning of the cellular response to RTK-inducing signals, members of the Spry family are good candidates for an important role in the tumorigenesis of different cells. Accordingly, Spry2 and/or Spry4 are shown to act as tumor-suppressors in cancer originated from, e.g., lung [14,15,16], liver [17], breast [18,19], prostate [20] and bone [21]. In other types of tumors, members of the Spry protein family fulfill a tumor-promoting task as it was demonstrated for Spry2 in colon carcinoma [22,23] and for Spry1 in rhabdomyosarcoma [24]. In brain tumors, repression of Spry2 has been shown to interfere with proliferation of GBM-derived cell lines and tumor formation [25,26]. Compatible with the tumor-promoting function of Spry2 in brain, the Spry proteins are important for other neuronal processes. Spry2 as well as Spry4 downregulation is associated with promoted axon outgrowth [27,28], and Spry1, Spry2 and Spry4 inhibit FGF-induced processes in the cerebellum [29]. Data generated in *Xenopus* document that Spry3 is important in regulating axon branching of motoneurons [30], and the finding that Spry3 is associated with autism susceptibility indicates a further role in the human brain [7].

In the presented study, we investigated the expression of Spry3 and Spry4 in brain cancer-derived cells and analyzed how a modulation of their expression influences the behavior of glioblastoma-derived cell lines.

## 2. Material and Methods

### 2.1. Cell Lines

The astrocytoma-derived cells (SW1088) and both neuroblastoma-derived cell lines (SK-N-DZ and SK-N-FI), as well as the glioblastoma-derived cell lines DBTRG-05MG, T98G and U373 and the oligodendroglioma-derived cell line Hs683 were purchased from the American Type Culture Collection (ATCC). NMC-G1, a cell line established from an astrocytoma, and AM-38, a glioblastoma originated cell line, were obtained from the JCRB cell bank. Cell lines LN40 and LN140 were kindly provided by Dr. Tribolet (Lausanne). Cell lines BTL1529, BTL2177 and BTL53 were established from glioblastoma diagnosed patients and BTL1376 and BTL2175 from gliosarcoma patients at the Neuromed Campus in Linz (NML) as described [31]. The cell line VBT72 was established from a glioblastoma at the Institute for Cancer Research [31]. These cell lines were kindly provided by Walter Berger (Medical University of Vienna). All cells were cultured in the recommended medium containing 10% fetal calf serum (FCS) and supplemented with penicillin (100 U/mL) and streptomycin (100 µg/mL) at 37 °C in 7.5% CO_2_.

### 2.2. Adenoviral Infection of Cells

The coding sequence of human Spry3 was amplified by PCR using Pfx Polymerase (Invitrogen) with upstream primer 5-AGCTCTGGATCCATGGATGCTGCGGTGACAGAT-3 (Spry3-s) and downstream primer 5-TAGCGAATTCCTCGAGTCATACAGACTTT-3 (Spry2-as) to add appropriate cloning sites. The amplified DNA fragments were subsequently cloned via BamHI/EcoRI into a pADlox plasmid to generate pADlox-Spry3. To construct an adenovirus expressing shRNA directed against Spry3, the CMV promoter of pADlox was exchanged by the human U6 promoter of the pSilencer Vector. Two oligonucleotides harboring an shRNA directed against Spry3 were annealed: sh-Spry3 sense 5′-TCG AGC GCA GCT GTT CAA TAG GCA GAA TTT GTT GAA GCT TGA ACA AAT TCT GCC TAT TGA ACA GCT GCG CTC TTT TTT-3′ and shSpry3 as 5′-AAT TAA AAA AGA GCG CAG CTG TTC AAT AGG CAG AAT TTG TTC AAG CTT CAA CAA ATT CTG CCT ATT GAA CAG CTG CGC-3′. The double stranded DNA with overlapping XhoI and EcoRI sites was then inserted in the digested pAdloxU6 vector to obtain pADlox-shSpry3. To obtain a virus directed against Spry4, two oligonucleotides (5′-TCGAGCTCAGCTCGCTACCTCCGCGGCGATGTTGAAGCTTGAACATCGCCGCGGAGGTAGCGAGCTGAGCTGTTTTTT-3′ and 5′-AATTAAAAAACAGCTCAGCTCGCTACCTCCGCGGCGATGTTCAAGCTTCAACATCGCCGCGGAGGTAGCGAGCTGAGC-3′) were annealed and subcloned the same way to construct pADlox-shSpry4. The correct cloning was confirmed by sequencing analysis. Recombinant viruses were produced as described [32]. Adenoviruses expressing Spry4 or control proteins (luciferase, lacZ or CFP) were already generated [21,33].

The optimal concentrations of the viruses for each cell line was determined by infecting the cells with different dilution of adenoviruses expressing Cyan Fluorescence Protein (CFP). The viral concentration of the adenoviruses expressing different proteins were calculated according to their OD_260_. For infection, viruses were diluted in serum-free medium.

### 2.3. Cell Signaling Assay

For analyzing ERK phosphorylation, 10^5^ cells were seeded into Ø6 cm tissue culture plates in DMEM medium containing 10% FCS. Twenty-four hours later, the cells were washed with and incubated in serum-free medium. Next day cells were infected with adenoviruses and incubated for another 2 days before 20% FCS or 10ng/mL FGF2 were added.

### 2.4. Scratch Assay

For the scratch assay, 6 × 10^5^ cells were infected with adenoviruses expressing the control proteins, Spry3 or Spry4, respectively. A total of 24 h post infection, cells were transferred into a 6-well plate. The next day, three straight scratches per well were introduced into the monolayer using a sterile yellow pipette tip. To remove debris, cells were washed twice with 1 x PBS. Finally, 3 mL of DMEM supplemented with 10% FCS were added. The closing of the scratch was pictured by the VISITRON Live Cell Imaging System (Visitron, Puchheim, Germany) at 10x magnification using VisiView Software. The running time was set to 40 h and for monitoring a time interval of 30 min was chosen. Using ImageJ software, gap width of three scratches were calculated every two hours. Migration velocity was assessed by applying linear regression using GraphPad Prism software. Migration velocity of three independent experiments were compared.

### 2.5. Growth Curve

Growth curves were performed and analyzed as described [21]. Each growth curve was counted in triplicate and after assessing the continuity of the growth by depicting it in a semi-logarithmical graph, the doubling time was calculated by applying an exponential growth equation. The calculated doubling times of at least three independent experiments were compared to each other and differences between two groups were calculated using an unpaired t-test.

### 2.6. Immunoblot

Immunoblotting was carried out as described [34]. The antisera against Spry4 and Spry3 were produced and affinity-purified as described [15]. The Spry3 antibodies were raised against the N-terminal 200 amino acids of the human homolog. As a loading control, antibodies against GAPDH (sc-365062) and ERK 1/2 (sc-514302) were purchased from Santa Cruz. Antibodies against phosphorylated extracellular signal-regulated kinase (pERK) (#9101) were received from Cell Signaling Technology. The horseradisch peroxidase-coupled secondary antibodies were purchased from GE Healthcare.

## 3. Results

### 3.1. In Brain Cancer-Derived Cell Lines Spry3 Protein is Commonly Expressed Independent of Mitogen Availability

**Figure 1:**
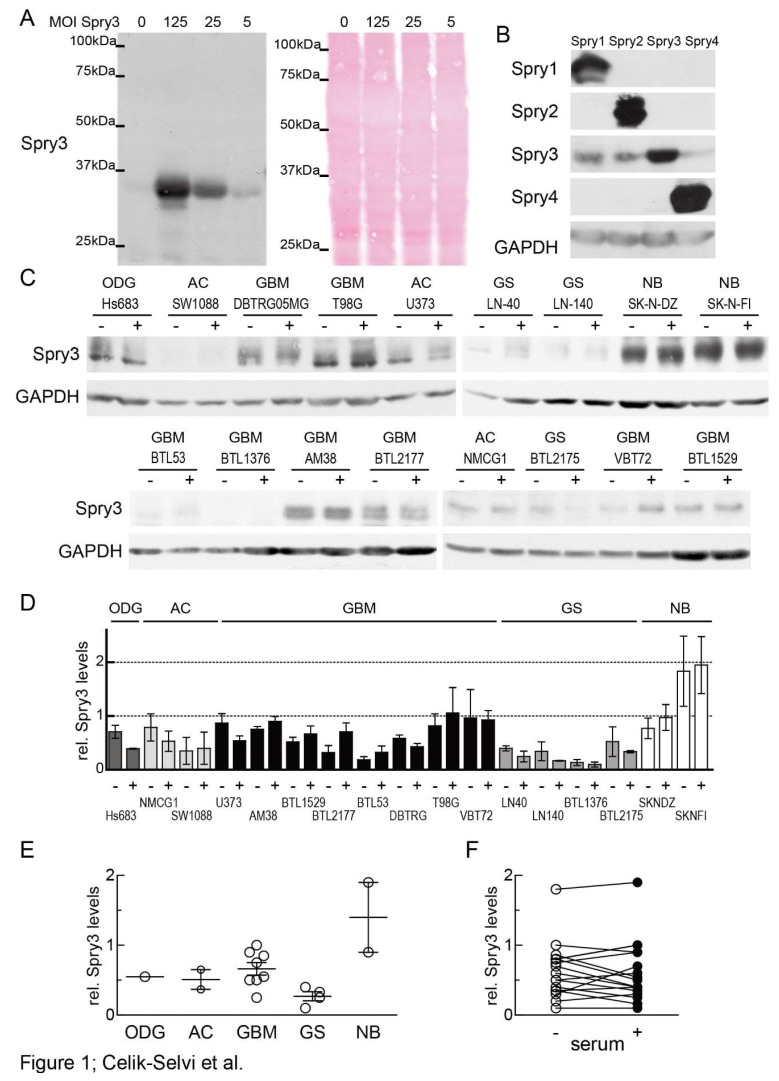
Expression of Spry3 protein in brain cancer-derived cell lines. (**A**) U373 cells were infected with decreasing amounts of adenoviruses expressing Spry3 protein and an immunoblot using Spry3 antibodies was performed. Equal loading was verified by Ponceau S staining of the immunoblot. (**B**) Adenoviruses expressing Spry1, Spry2, Spry3 or Spry4 were introduced into U373 cells. A total of 48 h post-infection cells were harvested and proteins were isolated. An immunoblot sequentially probed with all of the indicated antibodies is depicted. (**C**) Logarithmically growing cell lines derived from oligodendroglioma (ODG), astrocytoma (AC), glioblastoma (GBM), gliosarcoma (GS) and neuroblastoma (NB) were cultured for 24 h without (-) and with (+) serum. Using Western blot, endogenous Spry3 and GAPDH proteins were determined. (**D**) Amounts of Spry3 proteins were measured as ratio to an external control (MG63) by Image Quant software and normalized to GAPDH. Quantification results of 2–3 Western blots depicted as mean ± SEM are shown in a column graph. Cell lines were sorted according to their histopathological origin. (**E**) A scatterplot presenting the Spry3 expression across the histopathological subgroups of brain cancer is shown. (**F**) Calculated Spry3 levels from cells grown in serum-deprived (open circle) and –supplemented (closed circle) mediums are compared.

First, we investigated if Spry3 expression is influenced by the grade of malignancy or the histological background of brain cancer-derived cells. In order to analyze Spry3 protein levels, antibodies had to be produced, affinity-purified and their sensitivity as well as their specificity had to be assessed. To analyze their sensitivity, U373 cells were infected with decreasing amounts of adenovirus expressing Spry3 protein. As depicted in Figure 1A, the antibodies detected a single band at 33 kDa and in cells infected with decreasing titers of Spry3-encoding adenoviruses, the intensity of the detected band corresponded to the amount of introduced viruses while the cellular protein content was comparable. To control the specificity, all four Spry proteins were ectopically expressed by using the respective adenoviruses. Two days after infection, sufficient amounts of all Spry proteins were expressed, but the Spry3 antibody only detected Spry3 (Figure 1B). In the subsequent experiment, we determined the endogenous levels of Spry3 in different brain cancer-derived cell lines. To analyze if, like it was shown for Spry2 and Spry4 [35], Spry3 protein expression is dependent on mitogens in the cellular environment, serum was withdrawn from part of the cells (-), and their Spry3 levels were compared to those of cells cultivated in the presence of serum (+). In only 1/17 cell lines Spry3 protein was undetectable. Most of the cell lines express detectable amounts of Spry3 proteins which appear in a distinguishable pattern of bands. Usually the slower migrating bands were more abundant in the presence of serum indicating that a serum-dependent modification is applied to Spry3 (Figure 1C). Concerning the influence of the histopathologic origin, we observed that in the more advanced GBM-derived cell lines the expression of Spry3 was on average higher than in cells originated from the lower graded ODG and AC (Figure 1D,E). The highest expression of Spry3 was detected in the two NB-derived bone morrow metastases. These observations would favor rather an oncogenic than a tumor-suppressing function of Spry3 in brain cancers. Interestingly, the serum had not the expected influence on Spry3 expression, as half of the cell lines failed to adapt their Spry3 expression in response to mitogen availability. In five of the cell lines, its expression even slightly increased (less than 2-fold) if serum was withdrawn. A more pronounced change of Spry3 in response to serum in form of an increase or decrease was only observed in one cell line each (Figure 1D,F). Therefore, it is unlikely that mitogen-induced signals play an important role in regulating the expression of Spry3.

### 3.2. Spry4 Protein Expression is Repressed in Cell Lines Derived from More Malignant Brain Tumors, but Usually Still Serum-Dependent

**Figure 2:**
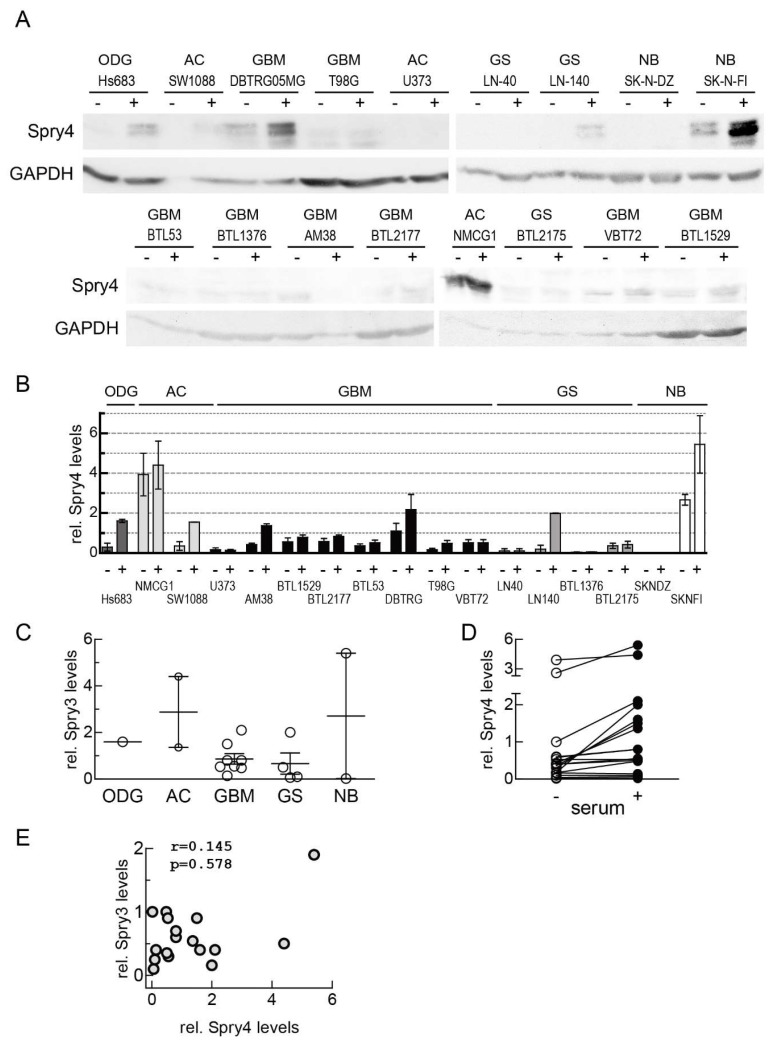
Expression analysis of endogenous Spry4 protein in brain cancer cells. (**A**) Spry4 protein levels in 17 brain cancer-derived cell lines which were cultured devoid of (−) and with (+) serum. GAPDH served as loading control. (**B**) Quantification of Spry4 was performed using Image Quant 5.0. An external control was arbitrarily set as 1 and loading differences were adjusted to GAPDH expression. A column graph summarizes the results of 2–3 independent experiments. (**C**) Spry4 expression in serum-supplemented growth condition was compared. A scattered dot-plot grouping the cells according to the histological origin is depicted. (**D**) A comparison of Spry4 levels detected in starved (open circle) and stimulated (closed circle) cells is presented. (**E**) Correlation of Spry3 and Spry4 expression was calculated using GraphPad Prism.

In order to investigate that growth factor-induced signaling in the analyzed cell lines is able to sufficiently influence the negative feedback loop responsible for controlling Spry protein expression, Spry4 protein levels were determined in comparison. In some cell lines, Spry4 expression was very prominent, but in five of them, we were not able to detect Spry4 proteins. Like in the case of Spry3, Spry4 frequently appeared in more than one migrating form (Figure 2A). Compared to the levels detected in cells derived from lower graded patients’ tissues, usually Spry4 expression in GBM and GS is strongly repressed, although in few of these cell lines Spry4 protein was definitively abundant (Figure 2B,C). Only in five of the cells lines the expression of Spry4 in serum-free conditions was insignificantly changed when compared to the parallel in serum cultivated cell counterparts. Seven of the brain-derived cell lines displayed a more than twofold decrease of Spry4 protein as a consequence of serum starvation. Moreover, in three of them the detected difference between the serum and non-serum condition exceeded a fivefold dimension (Figure 2B,D). When Spry3 and Spry4 expression in the different brain-derived cell lines were compared (Figure 2E), we found that there was no correlation indicating that Spry3 and Spry4 expression are regulated by independent mechanisms.

### 3.3. MAPK Activation in Response to FGF and Serum is Effectively Inhibited by Spry4 While Spry3 Failed to Fulfill This Function in the Presence of Serum

**Figure 3:**
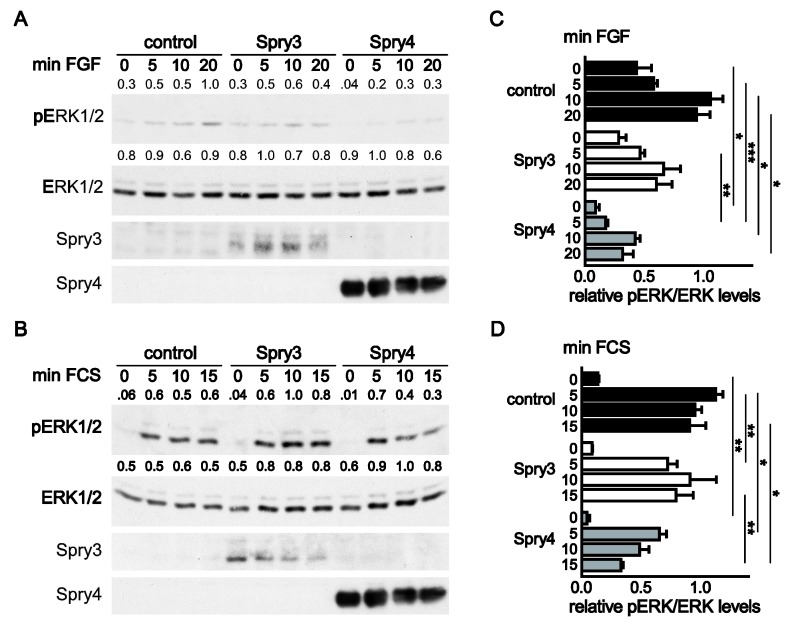
Influence of Spry3 and Spry4 proteins on ERK activation by FGF2 and serum. Glioblastoma (GBM)-derived cells (U373) were serum-starved for 24 h and then infected with adenoviruses expressing either a control protein (luciferase), Spry3 or Spry4. Two days later, cells were incubated with FGF2 (**A**) or serum (**B**) for the indicated times. Representative immunoblots of an experiment using antibodies recognizing pERK1/2 and total ERK1/2 are shown. Expression of Spry3 and Spry4 were verified by the respective antibodies. Using ImageQuant 5.0, the pERK1/2 bands detected were quantified and normalized to the corresponding values obtained for the ERK expression. The highest values were arbitrarily set as 1. The results of the quantification for the presented blots are depicted. (**C**) A summary of calculated mean values ± SEM of the pERK/ERK values from three experiments using FGF2 to stimulate the cells is depicted. Significance between the three groups was calculated by using a one-way ANOVA test in GraphPad prism. (**D**) The bands of pERK and ERK in response to serum were densitometrically quantified using ImageQuant 5.0, and the highest values of each experiment were set as 1. The graph summarizes three experiments. Significance was determined by a one-way ANOVA test in using GraphPad prism software. * *p* < 0.05; ** *p* < 0.01; *** *p* < 0.001.

To analyze if Spry3 and Spry4 are able to interfere with FGF-induced signaling, U373 cells were serum-deprived for 3 days before FGF2 was added for 5, 10 and 20 min. Within the starvation period a portion of cells were infected with viruses expressing Spry3, Spry4 or a control protein. In control treated cells, adding of FGF induced the MAPK pathway after 10 min as measured by determining the fraction of pERK (Figure 3A). In cells expressing excessive amounts of Spry3, like in the control cells, activation of the MAPK pathway was also observed after 10 min, but the extent of phosphorylation was less pronounced (Figure 3A,C). In case of ectopic Spry4 expression we detected that the proportion of activated ERK in serum-starved conditions was clearly less distinct. The addition of FGF caused an augmentation of the pERK levels, which was less intense than in the other two groups (Figure 3A,C). These data evince the inhibitory role of Spry proteins on FGF-mediated signaling, but demonstrated that Spry4 was more potent concerning interference with MAPK activation than Spry3.

In order to asses if the two Spry forms differ concerning their potential to inhibit MAPK activation in response to serum, a respective cell signaling assay was applied. In response to serum, ERK was immediately phosphorylated to a much higher extent (at least 10 times the value observed in starved cells) than in FGF-treated cells (two- to threefold induction). When Spry3 was expressed, the induction was slightly delayed but the amplitude was not significantly diminished. In contrast, Spry4 inhibited ERK phosphorylation significantly. As already observed in case of FGF induction, the basal pERK levels of cells cultivated in the absence of mitogens was clearly diminished, but also the maximal levels of pERK phosphorylation were reduced in comparison to the cells expressing a control or Spry3 protein. These data demonstrate that Spry4 can potently interfere with induction of the MAPK and indicate that Spry4 was more potent concerning interference with MAPK activation than Spry3.

### 3.4. In Brain Cancer-Derived Cell Lines, Spry3 and Spry4 Expressions Have an Opposing Effect on Cell Proliferation

**Figure 4:**
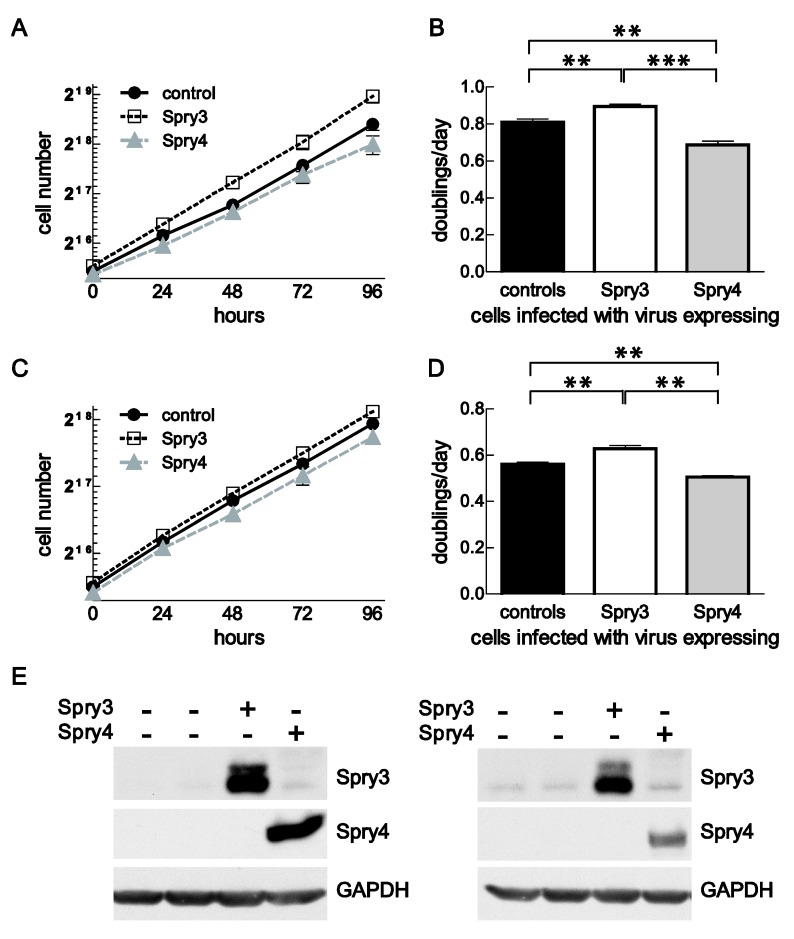
Influence of ectopic Spry3 and Spry4 expression on cell proliferation. Proliferation of cells overexpressing the indicated proteins was assessed by growth curve analysis. (**A**) The number of DBTRG-05MG cells were counted every 24 h for 5 days and are depicted as growth curves using a semi-logarithmical scale. A representative growth curve of three replicates is depicted. (**B**) Using GraphPad Prism, doubling times of at least three independent growth curve analyses performed with DBTRG-05MG cells were calculated and presented as mean doublings per day ± SEM. (**C**) A representative growth curve of U373 cell line is shown. (**D**) Using exponential growth equations, doubling times of U373MG cells were calculated and shown as doublings per day. Significance was assessed using an unpaired t-test in GraphPad Prism and mean ± SEM are shown. * *p* < 0.05; ** *p* < 0.01; *** *p* < 0.001 (**E**) Overexpression of Spry3 and Spry4 in the GBM cell lines DBTRG-05MG (left) and U373 (right) were verified by immunoblotting.

To investigate if Spry3 and Spry4 interfere with cell proliferation in brain cancer-derived cells, we selected DBTRG-05MG and U373 cell lines to apply ectopic overexpression of the respective Spry proteins. Both of these cell lines were easy to infect by adenoviruses as tested by using CFP expressing adenoviruses (data not shown). Furthermore, in DBTRG-05MG Spry3 appears mainly in its slower migrating form and Spry4 levels are pronounced while in U373 Spry3 mainly appears in its faster migrating form and a shift is only detected after serum addition. Spry4 was not detected in this cell line (Figure 1C and Figure 2A). To measure cell proliferation, growth curve analyses were performed. In DBTRG-05MG, Spry3 expressing cells double significantly faster (0.9 ± 0.01 doublings per day) than control treated cells (0.8 ± 0.02) while Spry4 expression decelerate the proliferation process to only 0.69 doublings per day (Figure 4A,B). Corroborating in U373, Spry3 accelerate cell proliferation from 0.56 ± 0.01 to 0.63 ± 0.01 doublings per day and Spry4 expression inhibits cell expansion to 0.51 ± 0.01 (Figure 4C,D). In both cell lines, Spry3 and Spry4 proteins are clearly overexpressed if the respective adenoviruses are applied (Figure 4E).

These data demonstrate that cell proliferation is promoted by Spry3 and suppressed by Spry4 expression arguing for an opposing effect of these Spry members.

### 3.5. Spry3 and Spry4 Exert a Contrary Effect on the Migratory Capabilities of Brain Cells

**Figure 5:**
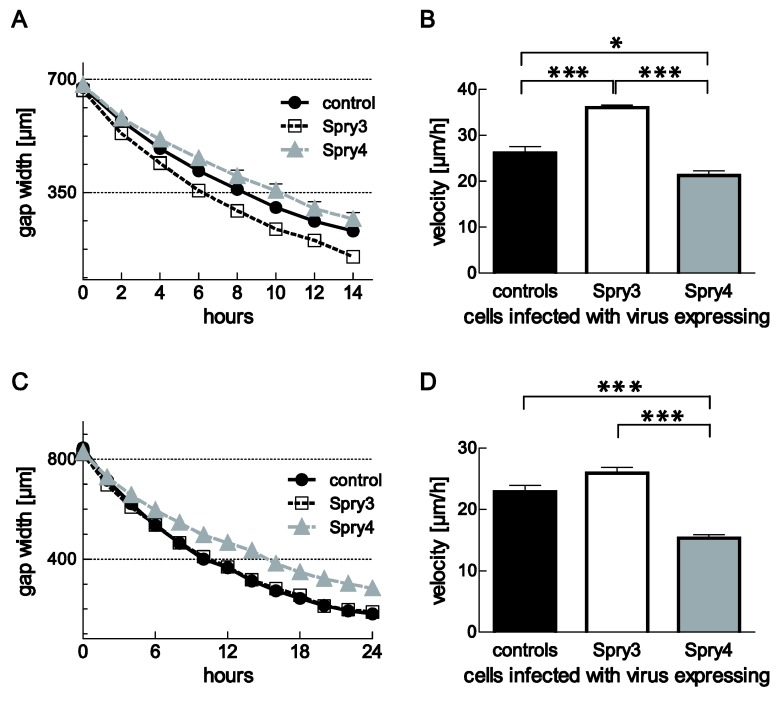
Influence of Spry3 and Spry4 expression on cell migration of GBM-derived cell lines. (**A**) Scratch assay was performed in DBTRG-05MG cells infected with adenoviruses expressing the indicated proteins. Representative curves of distance coverage were obtained by measuring decreasing gap widths of three replicative scratches at every two-hour time points using ImageJ. (**B**) Using linear regression, migration velocities were calculated. Means of at least three experimentations ± SEM are summarized as column bars. (**C**) Representative measurements of replicative gap closure in a close layer of U-373 MG cell expressing the indicated proteins are shown. (**D**) Velocities of at least three experiments were calculated using linear regression in GraphPad Prism and summarized in a graph depicting means ± SEM. An unpaired t-test was used to acquire significance. *p* < 0.05; ** *p* < 0.01; *** *p* < 0.001.

Aberrant cell migration is another RTK-mediated process contributing to the malignancy of cancer cells. Therefore, we next investigated if the expression of Spry3 and Spry4 proteins modulate the closure of the gap in a scratch assay. In DBTRG-05MG, ectopic expression of the Spry3 protein has a prominent influence on cell migration by augmenting their velocity from 26.1 ± 1.4 to 36.1 ± 0.5 µm/h. In contrast, Spry4 expression slows down these cells to 21.3 ± 0.99 µm/h (Figure 5A,B). Both effects were significant.

Compared to DBTRG-05MG, U373 cells are slower migrating and the effect of the Spry proteins was less developed. Spry3 has no significant effect on the velocity of gap closure although a slightly faster calculated average velocity points towards a positive effect of its expression on cell migration. In accordance with the data obtained in DBTRG-05MG, Spry4 expression in U373 delays the closure of the gap significantly. The control-treated cells move with a speed of 22.9 ± 1.0 µm/h towards the opposite front, while in the presence of Spry4 as an average speed only 15.4 ± 0.5 µm/h were calculated (Figure 5C,D).

These observations indicate that in brain cells, Spry3 and Spry4 exert different effects not only on cell proliferation but also on cell migration.

### 3.6. Repressed Expression of Spry3 Inhibits Cell Proliferation and Migration

**Figure 6:**
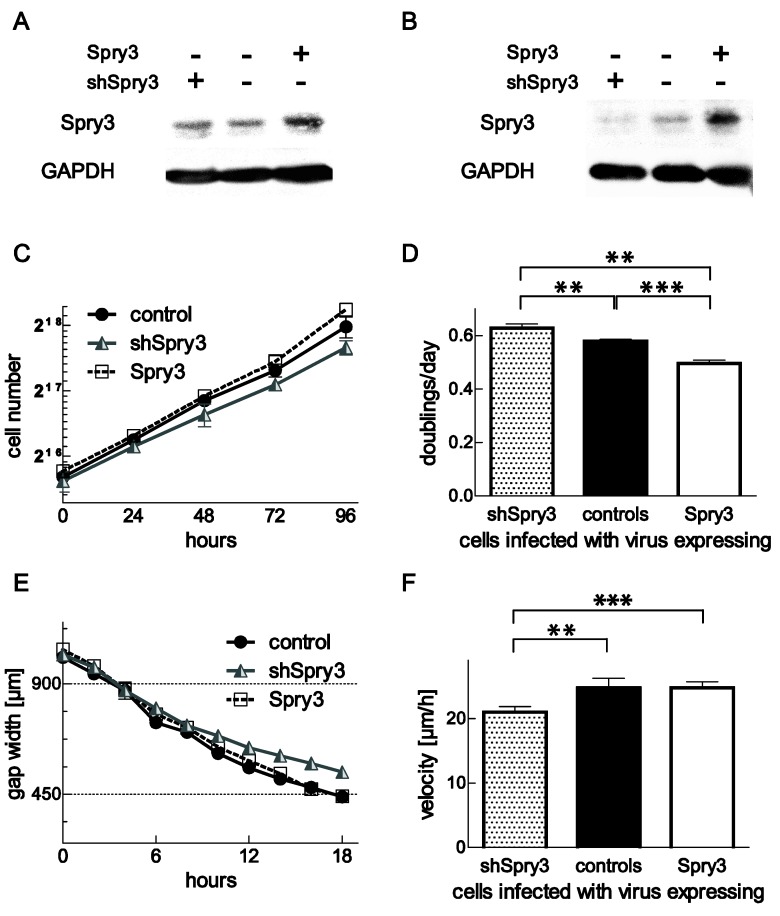
Verification of Spry3 impact on cell proliferation and migration by downregulation of the endogenous protein levels. DBTRG-05MG (**A**) and U373 (**B**) cells infected with adenoviruses expressing Spry3, shSpry3 or a control protein were analyzed concerning their Spry3 protein levels. (**C**) Three days after infection with the indicated viruses a growth curve analysis was performed in U373. (**D**) The doubling time of three experiments was calculated by performing an exponential growth equation and the mean doublings per day ± SEM are depicted. (**E**) U373 cell expressing the indicated proteins were cultured to form a close layer before a scratch assay was performed. Measurements of three replicative gaps were performed every two hours and a representative experiment is shown. (**F**) Velocities of three experiments were calculated using linear regression in GraphPad Prism and a summary is depicted. Using an unpaired t-test, significance was determined. ** *p* < 0.01; *** *p* < 0.001.

To further verify our observations, we wanted to investigate if lowered Spry expressions would influence cell proliferation in the opposite way than their overexpression. Therefore, an adenovirus expressing a shRNA directed against Spry3 was introduced into DBTRG-05MG and Spry3 levels were compared to the ones in control-treated and Spry3 overexpressing cells. As depicted in Figure 6A, expression of shSpry3 failed to modulate Spry3 levels, while the overexpression was successfully applied. In contrast, in U373 cells, expression of shRNA targeting Spry3 mRNA resulted in clearly lowered levels of Spry3 protein (Figure 6B). Since Spry4 is not expressed in detectable amounts in U373, it was just useful to express shSpry4 in DBTRG-05MG, where similar to the application of shSpry3, the endogenous expression of the protein was unaffected by expressing a shRNA directed against Spry4 (data not shown). Next, we performed a growth curve analysis using shSpry3 in U373 cells. Reduced Spry3 expression caused an inhibition of cell proliferation while in parallel overexpression accelerated the doubling of these cells (Figure 6C). Compared to control cells, doubling of shSpry3-treated cells was reduced from 0.58 to 0.50 doublings per day, substantiating an oncogenic effect of Spry3 in brain cancer (Figure 6D). To evaluate if a repression of Spry3 in addition to its interference with proliferation is also influencing cell migration, Spry 3 levels of U373 cells were modulated by treatment with the respective adenoviruses and a scratch assay was performed. The time to close the gap was significantly delayed when Spry3 levels were lowered (Figure 6E). On average, cells expressing a shRNA targeting Spry3 cover a distance of 21 µm in an hour, while control treated cells move about 1.2-fold faster, while Spry3 overexpression had no significant influence on the velocity of gap closure (Figure 6F). These data demonstrate that like proliferation, cell migration of GBM-derived cells is hindered if less Spry3 proteins are present confirming the tumor-promoting function of Spry3.

## 4. Discussion

Deregulated signal transduction is one of the most frequent alterations contributing to malignancy of brain cancer. In this study we provide data showing that Spry3 and Spry4 expression may be altered in brain cancer and affect cell proliferation and migration in opposing ways. Both Spry proteins are expressed in most of the brain cancer-derived cell lines, and two bands with a slightly different migration velocity can be detected. In case of Spry3, the faster migrating band is more frequently detected in the cells cultivated in the absence of serum. Although posttranslational modification of Spry3 is not reported, it is likely that analog to the other family members [36] the protein is phosphorylated at serine and/or tyrosine residues and that one of these potential modification is causing a shift in the gel.

A comparison of the Spry3 and Spry4 protein levels in the different cell lines revealed that the expression of these two Spry family members does not correlate. While Spry3 expression was on average elevated in cell lines originated from higher malignant tumors, Spry4 tended to be repressed in GBM and GS compared to cells derived from lower graded cancers. In accordance with our observation, an earlier report describes that Spry4 is often missing or deleted in gliomas [37]. Indeed, in several of the GBMs analyzed we were unable to detect this Spry isoform. Similarly, in lung [14,38] and breast cancer [39], a repression of Spry4 is coinciding with a postulated tumor-suppressive function. With regard to Spry3, in normal brain tissue its expression is well documented, but due to its low abundance in other tissues, expression data in cancers are rarely available [6,7]. To our knowledge, only one report by Sirivatanauksorn et al. investigated the RNA level of Spry3 as well as Spry4 in hepatocellular cancer, and comparable to our observations in the brain, Spry4 mRNA levels were downregulated in liver cancer-associated tissue, while Spry3 expression was unaltered [40]. With respect to Spry2, data generated on RNA level clearly points towards an upregulation of this Spry member in GBM when compared to non-tumor tissue [25], while a study exploring protein levels in immunohistochemistry suggests downregulation of Spry2 in higher malignant brain cancers when compared to lower graded tumors [41]. Another obvious different variable concerning regulation of Spry3 and Spry4 is their dependency on mitogen availability. Like Spry1 in lung cancer cells [34], Spry3 protein levels fail to fluctuate in response to serum-withdrawal in brain cancer-derived cells. In contrast, Spry4 expression is usually manifold augmented when serum-containing factors are supplied. This is in accordance with observations in lung [35,42], prostate and osteosarcoma [42]. Additionally, it is reported that in neuronal cells derived from the dorsal root ganglion, Spry4 can be induced as a consequence of FGF2 as well as NFG supplementation [28]. Differences in Spry3 and Spry4 expression control are furthermore reported in bovine ovarian granulosa cells where Spry4 was increased in response to FGF1 and FGF4, while in parallel Spry3 levels were lowered [43].

Concerning their impact on the cellular behavior, we observed that ectopic expression of Spry3 is augmenting the growth and migration rate of different GBM-derived cell lines. Corroborating, repression of its protein levels as achieved by introducing a specific shRNA resulted in diminished cell proliferation. This would suggest that this Spry protein member exerts a tumor-promoting role in brain cancers. Accordingly, two different reports suggest that Spry2 is advantageous for the malignancy of GBM [25,26]. Knock-down of its expression decelerates cell proliferation of GBM cell lines [25,26] while astrocytes were unaffected by modulated Spry2 levels [25]. Additionally Spry2 was identified as prognostic marker for GBM patients survival [25]. Although in most tissues Spry proteins fulfill the function of tumor-suppressors, individual Spry proteins are promoting tumorigenic potential here and there [44]. Spry2, for example, is above its function in GBM, shown to promote colon cancer malignancy by increasing proliferation, migration, tumor growth [23] and invasion [22] of colon cancer cells. In case of Spry1, an oncogenic function of the protein was demonstrated in the embryonal subtype of rhabdomyosarcoma [24].

In contrast to Spry3 and Spry2, Spry4 expression is inhibiting cell migration and proliferation of GBM-derived cell lines and is able to inhibit ERK phosphorylation in FGF2- and serum-induced as well as in unstimulated GBM-derived cells. An opposing role of Spry4 to other Spry proteins is already signified in colon carcinomas. Zhou et al. [45] demonstrated that Spry4 expression interferes with in vitro and in vivo cell proliferation of colon cancer cells. In contrast, Spry2 and Spry1 are fulfilling oncogenic functions in these tumors [22,23,46]. Additionally, in osteosarcoma [21] and in ovarian cancer [47], a tumor-suppressing role for Spry2 but not Spry4 was explicitly highlighted. Nonetheless, cell migration is specifically inhibited by Spry4 expression in prostate [48], pancreatic [49] and endothelial cells [50]. A concordant inhibition of proliferation and migration in case of Spry4 expression is reported for breast [18] and lung cancer cells [14]. Additionally, Spry4 can fulfill a tumor-suppressing role by interfering with angiogenic signals and thereby inhibits neovascularization and tumor growth [51].

## 5. Conclusion

In summary, our study describes that Spry3 and Spry4 exert different roles in brain cancer. Spry3 potentiates the tumorigenic potential of glioblastoma cells and Spry4 functions as tumor-suppressing protein in this entity.
